# NADH dehydrogenase subunit-2 237 Leu/Met polymorphism modifies effects of cigarette smoking on risk of elevated levels of serum liver enzyme in male Japanese health check-up examinees: a cross-sectional study

**DOI:** 10.1186/1617-9625-12-11

**Published:** 2014-07-10

**Authors:** Akatsuki Kokaze, Masao Yoshida, Mamoru Ishikawa, Naomi Matsunaga, Kanae Karita, Tadahiro Ohtsu, Hirotaka Ochiai, Takako Shirasawa, Hinako Nanri, Yuta Baba, Hiromi Hoshino, Yutaka Takashima

**Affiliations:** 1 Department of Public Health, Showa University School of Medicine, 1-5-8 Hatanodai, Shinagawa-ku, Tokyo 142-8555, Japan; 2 Department of Public Health, Kyorin University School of Medicine, 6-20-2 Shinkawa, Mitaka-shi, Tokyo 181-8611, Japan; 3 Mito Red Cross Hospital, 3-12-48 Sannomaru, Mito-shi, Ibaraki 310-0011, Japan

**Keywords:** Alanine aminotransferase, Cigarette smoking, Gamma-glutamyltransferase, Mitochondrial DNA polymorphism, NADH dehydrogenase, Reactive oxygen species

## Abstract

**Background:**

NADH dehydrogenase subunit-2 237 leucine/methionine (ND2-237 Leu/Met) polymorphism reportedly influences the effects of cigarette smoking on respiratory function, risk of dyslipidemia, serum non-high-density lipoprotein cholesterol levels, hematological parameters and intraocular pressure. The objective of this study was to investigate whether ND2-237 Leu/Met polymorphism modifies the effects of cigarette smoking on serum liver enzyme levels in male Japanese health check-up examinees.

**Methods:**

A total of 421 male subjects (mean age ± SD, 54.1 ± 7.7 years) were selected from among individuals visiting the hospital for regular medical check-ups. After ND2-237 Leu/Met genotyping, a cross-sectional study assessing the combined effects of ND2-237 Leu/Met polymorphism and cigarette smoking on serum aspartate aminotransferase levels, serum alanine aminotransferase (ALT) levels and serum gamma-glutamyltransferase (GGT) levels was then conducted.

**Results:**

No statistically significant differences in serum liver enzyme levels among the three smoking status groups (never- or ex-smokers, 1–20 cigarettes smoked per day and >20 cigarettes smoked per day) by ND2-237 Leu/Met genotype were observed. However, for men with ND2-237Met, cigarette smoking significantly increased the risk of elevated levels of serum ALT (>30 U/L) or serum GGT (≥60 U/L or >51 U/L) (*P* for trend = 0.031, *P* for trend = 0.007 and *P* for trend = 0.004, respectively). After adjustment for age, body mass index, alcohol consumption, coffee consumption, antihypertensive treatment and antidiabetic treatment, a significant association between cigarette smoking and risk of elevated levels of serum ALT (>30 U/L) or serum GGT (≥60 U/L or >51 U/L) was also observed (*P* for trend = 0.032, *P* for trend = 0.019 and *P* for trend = 0.009, respectively). Surprisingly, for men with ND2-237Leu, cigarette smoking significantly decreased the risk of elevated levels of serum ALT (>30 U/L or ≥25 U/L) (*P* for trend = 0.026 and *P* for trend = 0.003, respectively).

**Conclusions:**

Cigarette smoking appears to increase the risk of elevated levels of serum ALT or serum GGT in ND2-237Met genotypic men, but to decrease the risk of elevated levels of serum ALT in ND2-237Leu genotypic men.

## Background

Hepatologists have not considered cigarette smoking to be a causative agent for chronic liver diseases [1]. However, cigarette smoking induces direct or indirect toxic effects, immunological effects and oncogenic effects on the liver [2]. There is increasing evidence that cigarette smoking deleteriously influences the incidence, severity or clinical course of many types of chronic liver disease [1]. Several epidemiological surveys [3-7] have reported that cigarette smoking is positively associated with serum liver enzyme levels, which have been clinically used as a biomarker for liver disease.

Mitochondrial DNA cytosine/adenine (Mt5178 C/A) polymorphism, also known as NADH dehydrogenase subunit-2 237 leucine/methionine (ND2-237 Leu/Met) polymorphism, is associated with longevity in the Japanese population [8]. The frequency of the ND2-237Met genotype is significantly higher in Japanese centenarians than in the general population [8]. Japanese individuals with ND2-237Met are more resistant to lifestyle-related adult-onset diseases, such as hypertension [9], diabetes [10], myocardial infarction [11,12] and cerebrovascular disorders [13], than those with ND2-237Leu. Moreover, ND2-237 Leu/Met polymorphism modulates the effects of cigarette smoking on respiratory function [14], the risk of dyslipidemia [15], serum non-high-density lipoprotein (non-HDL) cholesterol levels [16], hematological parameters [17] and intraocular pressure [18]. However, there has been no research on the interactive effects of ND2-237 Leu/Met polymorphism and cigarette smoking on serum liver enzyme levels; specifically, serum aspartate aminotransferase (AST) levels, serum alanine aminotransferase (ALT) levels or serum gamma-glutamyltransferase (GGT) levels.

The objective of this study is to investigate whether there is a joint effect of ND2-237 Leu/Met polymorphism and habitual smoking on serum liver enzyme levels in male Japanese health check-up examinees.

## Subjects and methods

### Subjects

Participants were recruited from among individuals visiting the Mito Red Cross Hospital for regular medical check-ups between August 1999 and August 2000. This study was conducted in accordance with the Declaration of Helsinki and was approved by the Ethics Committee of the Kyorin University School of Medicine. Written informed consent was obtained from 602 volunteers before participation. Because the number of women was insufficient for classification into groups based on ND2-237 Leu/Met genotype and cigarette smoking, female health check-up examinees were excluded. Male health check-up examinees with unclear data were also excluded. Thus, 442 men were enrolled in the study. Twenty-one individuals with a history of chronic liver diseases, including hepatitis B and hepatitis C, were also excluded. Therefore, subjects comprised 421 Japanese men (age, 54.1 ± 7.7 years; mean ± SD).

### Clinical characteristics of subjects

Blood chemical and physical data, including serum AST levels, serum ALT levels and serum GGT levels, in the present study were obtained from the results of regular medical check-ups [19]. Elevated levels of serum AST were defined as ≥30 U/L [20]. Elevated levels of serum ALT were defined as >30 U/L [21], or defined as ≥25 U/L [22]. Elevated levels of serum GGT were defined as ≥60 U/L [4-6] or >51 U/L [23,24]. Body mass index (BMI) was defined as the ratio of subject weight (kg) to the square of subject height (m). A survey of habitual smoking, alcohol consumption and coffee intake was performed by means of self-reported questionnaire. Smoking status was classified based on the number of cigarettes smoked per day (never- or ex-smokers; 1–20 cigarettes smoked per day; and >20 cigarettes smoked per day). Alcohol consumption was classified based on drinking frequency (non- or ex-drinkers; occasional drinkers, which include those who drink several times per week or per month; and daily drinkers). Coffee consumption was categorized based on the number of cups of coffee per day (less than one cup per day; one or two cups per day; and more than three cups per day). Information on antihypertensive treatment or antidiabetic treatment was derived from health check-up records.

### Genotyping

DNA was extracted from white blood cells using the DNA Extractor WB kit (Wako Pure Chemical Industries, Osaka, Japan). ND2-237 Leu/Met polymorphism was detected by polymerase chain reaction (PCR) and digestion with *Alu*I restriction enzyme. Primer sequences were: forward 5′-CTTAGCATACTCCTCAATTACCC-3′; and reverse 5′-GTGAATTCTTCGATAATGGCCCA-3′. PCR was performed with 50 ng of genomic DNA in buffer containing 0.2 μmol/l each primer, 1.25 mmol/l dNTPs, 1.5 mmol/l MgCl_2_ and 1 U of Taq DNA polymerase. After initial denaturation at 94°C for 5 min, PCR was conducted through 40 cycles in the following steps: denaturation at 94°C for 30 s, annealing at 60°C for 60 s and polymerase extension at 72°C for 90 s. After cycling, a final extension at 72°C for 10 min was performed. PCR products were digested with *Alu*I restriction enzyme (Nippon Gene, Tokyo, Japan) at 37°C overnight, and were electrophoresed on 1.5% agarose gels stained with ethidium bromide for visualization under ultraviolet light. The absence of an *Alu*I site was designated as Mt5178A (ND2-237Met), and the presence of this restriction site was designated as Mt5178C (ND2-237Leu).

### Statistical analyses

Statistical analyses were performed using SAS statistical software version 9.3 for Windows. Multiple logistic regression analysis was used to calculate odds ratios (ORs) for the risk of elevated levels of serum liver enzymes (AST: ≥30 U/L; ALT: >30 U/L or ≥25 U/L; GGT: ≥60 U/L or >51 U/L). For multiple logistic regression analysis and analysis of covariance, alcohol consumption (non- or ex-drinkers = 0, occasional drinkers, including those who drink several times per week or per month = 1, daily drinkers = 2), coffee consumption (<1 cup per day = 1, 1–2 cups per day = 2, ≥3 cups per day = 3), antihypertensive treatment (subjects without antihypertensive treatment = 0, those under antihypertensive treatment = 1) and antidiabetic treatment (subjects without antidiabetic treatment = 0, those under antidiabetic treatment = 1) were numerically coded. Differences with *P* values of less than 0.05 were considered to be statistically significant.

## Results

No significant differences in clinical characteristics, including serum liver enzyme levels, were observed between the ND2-237Leu and ND2-237Met genotypes (Table 1). For either definition of elevated levels of serum liver enzymes (AST: ≥30 U/L; ALT: >30 U/L or ≥25 U/L; GGT: ≥60 U/L or >51 U/L), no significant differences in the frequency of subjects with elevated levels of serum liver enzymes were observed between men with ND2-237Leu and those with ND2-237Met. Frequency of smoking status (never- or ex-smokers; 1–20 cigarettes smoked per day; and >20 cigarettes smoked per day) was not significantly different between the ND2-237Leu and ND2-237Met genotypes. Smoking duration was not significantly different between current smokers with ND2-237Leu and those with ND2-237Met. The lowest of smoking duration was 5 years in ND2-237Leu genotypic current smokers and was 9 years in ND2-237Met genotypic current smokers.

**Table 1:** Clinical characteristics of study subjects by ND2-237 Leu/Met genotype

	**ND2-237Leu**	**ND2-237Met**	***P *** **value**
	**N = 251**	**N = 170**	
Age (y)*	54.5 (7.6)	53.6 (7.8)	0.245
BMI (kg/m^2^)*	23.1 (2.8)	23.5 (2.6)	0.103
AST (U/L)***	22 (18–27)	23 (19–27)	0.687
Elevated levels of serum AST (≥30 U/L) (%)****	18.7	18.2	0.899
ALT (U/L)***	21 (15–32)	24 (18–35)	0.471
Elevated levels of serum ALT (>30 U/L) (%)****	27.1	30.6	0.436
Elevated levels of serum ALT (≥25 U/L) (%)****	37.9	45.9	0.100
GGT (U/L) ***	42 (27–75)	47 (30–75)	0.427
Elevated levels of serum GGT (≥60 U/L) (%)****	34.7	38.8	0.384
Elevated levels of serum GGT (>51 U/L) (%)****	39.4	45.9	0.189
SBP (mmHg)**	126.1 (16.0)	125.7 (13.9)	0.614
DBP (mmHg)**	73.9 (10.5)	73.5 (9.0)	0.628
T-C (mg/dL)*	204.2 (31.5)	202.5 (31.9)	0.590
LDL-C (mg/dL)*	122.5 (30.4)	118.4 (31.4)	0.182
HDL-C (mg/dL)**	55.0 (13.6)	55.9 (15.7)	0.576
TG (mg/dL)***	112 (84–156)	113 (81–161)	0.378
FPG (mg/dL)***	97 (92–105)	97 (91–103)	0.240
Smoking status (never- or ex-/1–20 cigarettes per day/>20 cigarettes per day) (%)	59.4/27.9/12.7	58.2/27.1/14.7	0.846
Alcohol consumption (non- or ex-/occasionally/daily) (%)****	17.9/34.3/47.8	12.9/40.0/47.1	0.285
Coffee consumption (<1 cup per day/1-2cups per day/≥3 cups per day) (%)****	45.8/31.5/22.7	36.5/40.0/23.5	0.120
Subjects under antihypertensive treatment (%)****	19.9	13.5	0.089
Subjects under antidiabetic treatment (%)****	7.2	7.7	0.855
	**Smokers with ND2-237Leu**	**Smokers with ND2-237Met**	
	**N = 102**	**N = 71**	
Smoking duration (y)*	33.5 (8.5)	32.4 (7.3)	0.352

BMI; body mass index, AST; aspartate aminotransferase, ALT; alanine aminotransferase, GGT; gamma-glutamyltransferase, SBP; systolic blood pressure, DBP; diastolic blood pressure, T-C; total cholesterol, LDL-C; low-density lipoprotein cholesterol, HDL-C; high-density lipoprotein cholesterol, TG; triglyceride, FPG; fasting plasma glucose.

Age, BMI, SBP, DBP, T-C levels, LDL-C levels, HDL-C levels and smoking duration are given as means (S.D.). GGT levels, AST levels, ALT levels, TG levels and FPG levels are given as median (interquartile range). *Student’s *t*-test, **Welch’s *t*-test, ***Mann–Whitney test, ****chi-squared test. All *P* values depict significance of differences between ND2-237Leu and ND2-237Met.

Tukey-Kramer correction for multiple comparisons revealed no statistically significant differences in serum AST, ALT or GGT levels in the three smoking status groups (never- or ex-smokers, 1–20 cigarettes smoked per day and >20 cigarettes smoked per day) by ND2-237 Leu/Met genotype (Table 2). After adjusting for age, BMI, alcohol consumption, coffee consumption, antihypertensive treatment and antidiabetic treatment, no significant relationships between cigarette consumption and any serum liver enzyme levels were observed in either ND2-237Leu or ND2-237Met genotypic men.

**Table 2:** **Serum liver enzyme levels by smoking status and ND2-237 Leu/Met genotype**

	**Smoking status**			***P *** **for trend**
	**Never- or ex-smokers**	**1-20 cigarettes smoked per day**	**>20 cigarettes smoked per day**	
ND2-237Leu	N = 149	N = 70	N = 32	
AST (U/L)	24.5 (3.9)	23.2 (5.6)	46.0 (8.3)	0.071
AST (U/L)†	20.4 (7.0)	18.4 (8.2)	40.6 (10.2)	0.120
ALT (U/L)	26.7 (3.2)	24.8 (4.6)	37.9 (6.8)	0.290
ALT (U/L)†	23.8 (5.6)	21.9 (6.6)	35.5 (8.2)	0.282
GGT (U/L)	65.4 (6.3)	67.8 (9.2)	74.0 (13.6)	0.574
GGT (U/L)†	55.6 (10.8)	61.9 (12.6)	68.9 (15.8)	0.338
ND2-237Met	N = 99	N = 46	N = 25	
AST (U/L)	24.7 (1.3)	26.3 (2.0)	26.2 (2.7)	0.521
AST (U/L)†	19.9 (2.7)	24.0 (2.7)	21.4 (3.7)	0.313
ALT (U/L)	27.9 (2.3)	33.0 (3.4)	32.8 (4.6)	0.209
ALT (U/L)†	22.2 (4.6)	31.4 (4.7)	25.8 (6.3)	0.168
GGT (U/L)	56.4 (5.1)	70.4 (7.5)	67.6 (10.2)	0.163
GGT (U/L)†	46.9 (10.5)	64.1 (10.7)	54.6 (14.3)	0.232

AST; aspartate aminotransferase, ALT; alanine aminotransferase, GGT; gamma-glutamyltransferase.

†AST, †ALT and †GGT levels are given as least-square means (S.E) adjusted for age, body mass index, alcohol consumption, coffee consumption, antihypertensive treatment and antidiabetic treatment.

Tukey-Kramer correction for multiple comparisons was applied.

In the case of elevated levels of serum ALT, defined as serum ALT levels > 30 U/L, for subjects with ND2-237Leu, a negative and significant association between cigarette consumption and risk of elevated levels of serum ALT was observed (*P* for trend = 0.007) (Table 3). However, after adjusting for age, BMI, alcohol consumption, coffee consumption, antihypertensive treatment and antidiabetic treatment, that association disappeared. In the case of elevated levels of serum ALT, defined as serum ALT levels ≥ 25 U/L, for subjects with ND2-237Leu, a negative and significant association between cigarette consumption and risk of elevated levels of serum ALT was observed (*P* for trend = 0.003). The OR for elevated levels of serum ALT was significantly lower in subjects with ND2-237Leu who consumed >20 cigarettes per day than in never- or ex-smokers with ND2-237Leu (OR = 0.282, 95% confidence interval (CI): 0.110-0.726, *P* = 0.009). After the aforementioned adjustment, the negative and significant association between cigarette consumption and risk of elevated levels of serum ALT remained significant (*P* for trend = 0.010). The adjusted OR for elevated levels of serum ALT was significantly lower in subjects with ND2-237Leu who consumed >20 cigarettes per day than in never- or ex-smokers with ND2-237Leu (OR = 0.255, 95% CI: 0.084-0.776, *P* = 0.016). On the other hand, for subjects with ND2-237Met, in the case of elevated levels of serum ALT, defined as serum ALT levels > 30 U/L, the positive and significant association between cigarette consumption and risk of elevated levels of serum ALT was observed (*P* for trend = 0.031). The OR for elevated levels of serum ALT was significantly higher in subjects with ND2-237Met who consumed >20 cigarettes per day than in never- or ex-smokers with ND2-237Met (OR = 2.732, 95% CI: 1.104-6.762, *P* = 0.030). After the aforementioned adjustment, the positive and significant association between cigarette consumption and risk of elevated levels of serum ALT remained significant (*P* for trend = 0.032). The adjusted OR for elevated levels of serum ALT was significantly higher in subjects with ND2-237Met who consumed >20 cigarettes per day than in never- or ex-smokers with ND2-237Met (OR = 3.017, 95% CI: 1.064-8.560, *P* = 0.038). The risk of elevated levels of serum AST does not depend on cigarette consumption for either ND2-237Leu genotypic subjects or ND2-237Met genotypic subjects (data not shown).

**Table 3:** Odds ratios (ORs) and 95% confidence intervals (CIs) for elevated levels of serum alanine aminotransferase (ALT) by ND2-237 Leu/Met genotype and smoking status

**Genotype and number of cigarettes smoked daily**	**Frequency**		**OR (95% CI)**	**Adjusted OR**^**† **^**(95% CI)**
	**Normal levels of serum ALT**	**Elevated levels of serum ALT**		
	**serum ALT levels ≤ 30 U/L**	**serum ALT levels > 30 U/L**		
ND2-237Leu				
0 (never- or ex-smoker)	101 (67.8)	48 (32.2)	1 (reference)	1 (reference)
1-20	55 (78.6)	15 (21.4)	0.574 (0.295-1.117)	0.525 (0.237-1.166)
>20	27 (84.4)	5 (15.6)	0.390 (0.141-1.074)	0.462 (0.143-1.492)
			*P* for trend = 0.026	*P* for trend = 0.120
ND2-237Met				
0 (never- or ex-smoker)	74 (74.8)	25 (25.2)	1 (reference)	1 (reference)
1-20	31 (67.4)	15 (32.6)	1.432 (0.660-3.079)	1.826 (0.723-4.614)
>20	13 (52.0)	12 (48.0)	2.732 (1.104-6.762)*	3.017 (1.064-8.560)*
			*P* for trend = 0.031	*P* for trend = 0.032
	**serum ALT levels < 25 U/L**	**serum ALT levels ≥ 25 U/L**		
ND2-237Leu				
0 (never- or ex-smoker)	82 (55.0)	67 (45.0)	1 (reference)	1 (reference)
1-20	48 (68.6)	22 (31.4)	0.561 (0.308-1.021)	0.501 (0.248-1.010)
>20	26 (81.3)	6 (18.7)	0.282 (0.110-0.726)**	0.255 (0.084-0.776)*
			*P* for trend = 0.003	*P* for trend = 0.010
ND2-237Met				
0 (never- or ex-smoker)	54 (54.6)	45 (45.4)	1 (reference)	1 (reference)
1-20	28 (60.9)	18 (39.1)	0.771 (0.378-1.572)	0.828 (0.365-1.879)
>20	10 (40.0)	15 (60.0)	1.800 (0.737-4.395)	1.675 (0.633-4.435)
			*P* for trend = 0.408	*P* for trend = 0.459

^†^OR adjusted for age, body mass index, habitual alcohol consumption, coffee consumption, antihypertensive treatment and antidiabetic treatment.

*P < 0.05, **P < 0.01.

In the case of elevated levels of serum GGT, defined as serum GGT levels ≥ 60 U/L, for subjects with ND2-237Met, the risk of elevated levels of serum GGT may depend on cigarette consumption (*P* for trend = 0.007) (Table 4). The OR for elevated levels of serum GGT was significantly higher in subjects with ND2-237Met who consumed >20 cigarettes per day than in never- or ex-smokers with ND2-237Met (OR = 3.289, 95% CI: 1.330-8.138, *P* = 0.010). After adjusting for age, BMI, alcohol consumption, coffee consumption, antihypertensive treatment and antidiabetic treatment, the risk of elevated levels of serum GGT may also depend on cigarette consumption (*P* for trend = 0.019). After the aforementioned adjustment, the OR for elevated levels of serum GGT was significantly higher in subjects with ND2-237Met who consumed >20 cigarettes per day than in never- or ex-smokers with ND2-237Met (adjusted OR = 3.030, 95% CI: 1.160-7.918, *P* = 0.024). In the case of elevated levels of serum GGT, defined as serum GGT levels > 51 U/L, for subjects with ND2-237Met, the risk of elevated levels of serum GGT may depend on cigarette consumption (*P* for trend = 0.004). The OR for elevated levels of serum GGT was significantly higher in subjects with ND2-237Met who consumed >20 cigarettes per day than in never- or ex-smokers with ND2-237Met (OR = 3.561, 95% CI: 1.400-9.058, *P* = 0.008). After adjusting for age, BMI, alcohol consumption, coffee consumption, antihypertensive treatment and antidiabetic treatment, the risk of elevated levels of serum GGT may also depend on cigarette consumption (*P* for trend = 0.009). After the aforementioned adjustment, the OR for elevated levels of serum GGT was significantly higher in subjects with ND2-237Met who consumed >20 cigarettes per day than in never- or ex-smokers with ND2-237Met (adjusted OR = 3.304, 95% CI: 1.220-8.946, *P* = 0.019). On the other hand, the association between ND2-237Leu genotype and risk of elevated levels of serum GGT does not appear to depend on cigarette consumption.

**Table 4:** Odds ratios (ORs) and 95% confidence intervals (CIs) for elevated levels of serum gamma-glutamyltransferase (GGT) by ND2-237 Leu/Met genotype and smoking status

**Genotype and number of cigarettes smoked daily**	**Frequency**		**OR (95% CI)**	**Adjusted OR**^**† **^**(95% CI)**
	**Normal levels of serum GGT**	**Elevated levels of serum GGT**		
	**serum GGT levels < 60 U/L**	**serum GGT levels ≥ 60 U/L**		
ND2-237Leu				
0 (never- or ex-smoker)	96 (64.4)	53 (35.6)	1 (reference)	1 (reference)
1-20	46 (65.7)	24 (34.3)	0.945 (0.520-1.717)	1.142 (0.570-2.291)
>20	22 (68.7)	10 (31.3)	0.823 (0.363-1.868)	1.133 (0.406-3.158)
			*P* for trend = 0.648	*P* for trend = 0.772
ND2-237Met				
0 (never- or ex-smoker)	68 (68.7)	31 (31.3)	1 (reference)	1 (reference)
1-20	26 (56.5)	20 (43.5)	1.687 (0.820-3.471)	1.926 (0.852-4.351)
>20	10 (40.0)	15 (60.0)	3.289 (1.330-8.138)*	3.030 (1.160-7.918)*
			*P* for trend = 0.007	*P* for trend = 0.019
	**serum GGT levels ≤ 51 U/L**	**serum GGT levels > 51 U/L**		
ND2-237Leu				
0 (never- or ex-smoker)	87 (58.4)	62 (41.6)	1 (reference)	1 (reference)
1-20	43 (61.4)	27 (38.6)	0.881 (0.493-1.575)	1.128 (0.556-2.285)
>20	22 (68.7)	10 (31.3)	0.638 (0.282-1.442)	0.823 (0.297-2.280)
			*P* for trend = 0.288	*P* for trend = 0.855
ND2-237Met				
0 (never- or ex-smoker)	62 (62.6)	37 (37.4)	1 (reference)	1 (reference)
1-20	22 (47.8)	24 (52.2)	1.828 (0.901-3.709)	2.223 (0.994-4.969)
>20	8 (32.0)	17 (68.0)	3.561 (1.400-9.058)**	3.304 (1.220-8.946)*
			*P* for trend = 0.004	*P* for trend = 0.009

^†^OR adjusted for age, body mass index, habitual alcohol consumption, coffee consumption, antihypertensive treatment and antidiabetic treatment.

*P < 0.05, **P < 0.01.

## Discussion

In the present study, the combined effects of ND2-237 Leu/Met polymorphism and habitual smoking on the risk of elevated levels of serum ALT or GGT were observed in male Japanese health check-up examinees. To the best of our knowledge, this is a novel gene-environment interaction on elevated levels of serum ALT or GGT. For subjects with ND2-237Met, cigarette consumption may increase the risk of raised levels of serum ALT or GGT. On the other hand, for those with ND2-237Leu, cigarette smoking appears to decrease the risk of elevated levels of serum ALT.

Epidemiological approaches revealed that cigarette smoking raises serum GGT levels [7] and is positively associated with raised levels of serum ALT [3] or serum GGT [4-6]. However, genetic factors were not included as confounding factors or effect modifiers in either of the aforementioned studies. Contemplating the results of the present study, the inclusion of genetic information may contribute to future epidemiological surveys with regard to the effects of cigarette consumption on serum ALT or GGT levels or the risk of elevated levels of serum ALT or GGT.

A large-scale population-based prospective study found that elevated levels of serum ALT (>30 U/L in men and >19 U/L in women) are associated with liver disease mortality, and that elevated levels of serum GGT (>51 U/L in men and >33 U/L in women) are associated with not only liver disease mortality but also mortality from all causes, malignancy and diabetes [23]. Another large-scale prospective study showed that serum ALT and GGT activity predicts future development of metabolic syndrome [25]. A cross-sectional study showed the association between serum GGT activity and metabolic risk factors for cardiovascular disease (CVD) among male Japanese subjects [26]. Therefore, based on the prevention of not only liver damage but also metabolic syndrome or CVD, smoking cessation should be encouraged, particularly for ND2-237Met genotypic men.

Among ND2-237Met genotypic men, cigarette consumption may increase the risk of raised levels of serum ALT or serum GGT. Except for respiratory function [14], the effects of cigarette smoking on clinical or subclinical findings are more apparent in men with ND2-237Met than in those with ND2-237Leu [15-18]. Cigarette smoking increases the risk of hyper-low-density lipoprotein (LDL) cholesterolemia or that of hypertriglyceridemia in men with ND2-237Met [15]. Moreover, habitual smoking also increases serum non-HDL cholesterol levels in ND2-237Met genotypic men [16]. Considering our results together with hyper-LDL cholesterolemia, hypertriglyceridemia and high levels of non-HDL cholesterol as risk factors for atherosclerotic diseases [27], habitual smoking may offset the antiatherogenic properties of ND2-237Met genotype. Among ND2-237Leu genotypic men, although cigarette consumption appears to reduce the risk of raised levels of serum ALT, it increases the risk of chronic obstructive pulmonary disease [14]. Therefore, smoking cessation is also recommended for them.

The mechanisms of the joint effects of ND2-237 Leu/Met polymorphism and habitual smoking on the elevated levels of serum ALT or GGT presumably depend on the biochemical differences in response to some compounds in cigarette smoke between ND2-237Leu and ND2-237Met. NADH dehydrogenase is regarded as the major physiological site of reactive oxygen species (ROS) generation in mitochondria, and is itself a target of assault by ROS [28]. Extrapolation from animal models [29,30] to human subjects would suggest that ND2-237Met suppresses ROS production and/or protects NADH dehydrogenase itself from ROS. Cigarette smoke contains high concentrations of ROS, and is thought to activate endogenous sources of ROS [31]. Considering that serum GGT has potential as an early and sensitive biomarker of oxidative stress [32], cigarette smoking unexpectedly increases the risk of elevated levels of serum GGT in ND2-237Met genotypic men rather than in ND2-237Leu genotypic men. Surprisingly, habitual smoking apparently decreases the risk of elevated levels of serum ALT in men with ND2-237Leu. In any case, elucidating these mechanisms remains a matter for further pharmacological investigation.

Limited to the studies in which subjects were Japanese, Interleukin-1β -31 T/C polymorphism [33] or cytochrome P450 -3860 G/A polymorphism [34] influences the effects of cigarette smoking on the risk of hepatocellular carcinoma. Moreover, gluthathione S-transferase polymorphisms, which are associated with risk of CVD and diabetes, influence the effects of cigarette smoking on the development of non-alcoholic fatty liver disease in Japanese subjects [35]. Therefore, gene-gene or gene-gene-environment interactions with liver diseases, including elevated levels of serum ALT or GGT, should be investigated. Moreover, we focused on only one single nucleotide polymorphism (SNP) in a single gene, namely Mt5178 C/A polymorphism in mitochondrial DNA. With only a single SNP in a single gene, there is a very high chance of false-positive or false-negative findings. Therefore, utilization of an effective tool for sensitive and accurate SNP detection, for example an SNP detector [36], will be desirable for further molecular epidemiological research.

In addition to the lack of other genetic information, there are several crucial limitations in this study. First, as compared with other epidemiological surveys on the effects of cigarette smoking on serum liver enzyme levels [3-6], the sample size was very small. Second, we analyzed only a single population, and to avoid errors in molecular epidemiological survey, it is necessary to analyze two or more independent data sets. Third, although similar subject selections have been applied in our previous studies [9,14-19,37], selection bias cannot be ruled out. Fourth, as this was a cross-sectional study, and it can indicate causal links, it cannot establish valid causality. To overcome these limitations, a follow-up study with a larger study sample, including additional populations, is necessary. Fifth, we do not have any information on the last cigarette smoked prior to the blood sample collection; such information would be useful in interpreting the present results. Sixth, because of a lack of data on amounts of alcohol intake, the evaluation of habitual alcohol intake was based on the frequency of alcohol consumption. Alcohol consumption was considered to be a confounding factor in the statistical analysis of this study. Although we have also used this evaluation method in previous studies [9,16,37], the existence of joint effects between ND2-237 Leu/Met polymorphism and volume of alcohol intake on serum ALT or GGT levels warrants further investigation.

## Conclusion

The present molecular epidemiological study demonstrates that ND2-237 Leu/Met polymorphism modifies the effects of cigarette smoking on the risk of elevated levels of serum ALT or GGT in male Japanese health check-up examinees. Cigarette consumption may increase the risk of elevated levels of serum ALT or GGT in ND2-237Met genotypic men. Surprisingly, habitual smoking appears to lower the risk of elevated levels of serum ALT in ND2-237Leu genotypic men. This gene-environment interaction may help elucidate the pathophysiological mechanisms underlying the association between cigarette smoking and serum ALT or GGT levels.

## Abbreviations

ALT: Alanine aminotransferase; AST: Aspartate aminotransferase; BMI: Body mass index; CI: Confidence interval; CVD: Cardiovascular disease; GGT: Gamma-glutamyltranferase; LDL: Low-density lipoprotein; Mt5178 C/A: Mitochondrial DNA cytosine/adenine; ND2-237 Leu/Met: NADH dehydrogenase subunit-2 237 leucine/methionine; non-HDL: Non-high-density lipoprotein; OR: Odds ratio; PCR: Polymerase chain reaction; ROS: Reactive oxygen species; SNP: Single nucleotide polymorphism.

## Competing interests

The authors declare that they have no competing interests.

## Authors’ contributions

AK designed the study, carried out the epidemiological survey, carried out genotyping, analyzed the data, and drafted the manuscript; MY and KK carried out the epidemiological survey; MI collected the samples; NM assisted with genotyping; TO, HO, TS, HN, YB and HH assisted in data analysis and helped with interpreting the results; YT designed the study and carried out the epidemiological survey. All authors have read and approved the final manuscript.
